# The Utility and Limitations of the Transfibular Approach in Ankle Trauma Surgery

**DOI:** 10.1155/2014/234369

**Published:** 2014-10-30

**Authors:** Mustafa Yassin, Avraham Garti, Muhammad Khatib, Moshe Weisbrot, Uzi Ashkenazi, Edward Ram, Dror Robinson

**Affiliations:** ^1^Department of Orthopedics, Hasharon Hospital, Rabin Medical Center, Affiliated with the Sackler School of Medicine, Tel Aviv University, 4937211 Petah Tikva, Israel; ^2^Department of Surgery, Hasharon Hospital, Rabin Medical Center, Affiliated with the Sackler School of Medicine, Tel Aviv University, 4937211 Petah Tikva, Israel

## Abstract

The commonly used extensive approaches to the distal tibia include the posteromedial and anterolateral approaches. The current report describes several cases performed using this technique establishing a rationale and safe zone for performing a transfibular approach to the distal tibia. The advantages of such approach are the excellent visualization of the lateral tibia and the articular space. The utilization of this approach involves the risk of injury to the anterior tibial vessels and to the superficial peroneal nerve as well as a requirement for syndesmosis reconstruction. The recommendation is to utilize this approach in cases of severe comminution of the lateral tibia with a relatively intact medial tibia.

## 1. Introduction

There are different surgical approaches for fixing distal tibia fractures. The most commonly used anterior approach or alternatively a posteromedial or even a posterolateral approach may be utilized [1]. The anterior approach passes through the internervous plane between the superficial peroneal nerve and the deep peroneal nerve, while the posteromedial approach utilizes the interval between the saphenous nerve and the peroneal nerve. In rare cases of limb crush injury, an approach that does not compromise skin vitality in the anterior or medial aspects, such as the transfibular approach, is warranted. This approach is occasionally used in cases of distal tibia neoplasms such as enchondromas [2]. The transfibular approach to the distal tibia limits the risk to nerve damage by passing in an internervous plane. The plane is plane between the sural nerve and superficial peroneal nerve. This incision allows visualization, reduction, and fixation of both the posterior malleolus and the lateral malleolus via a single incision. As this approach necessitates release of the tibiofibular stabilization system, it is utilized only in cases with extensive traumatic damage to the syndesmosis. In most other cases, the syndesmosis is reconstructed at the end of the procedure. The current report describes 2 cases utilizing the transfibular approach in ankle fractures, an indication that to the best of our knowledge has not been previously described. In addition we present a cadaver study for a definition of safe zones in this type of surgery avoiding the deep peroneal nerve on its route along the interosseous membrane.

## 2. Case Report 1

**Figure 1:**
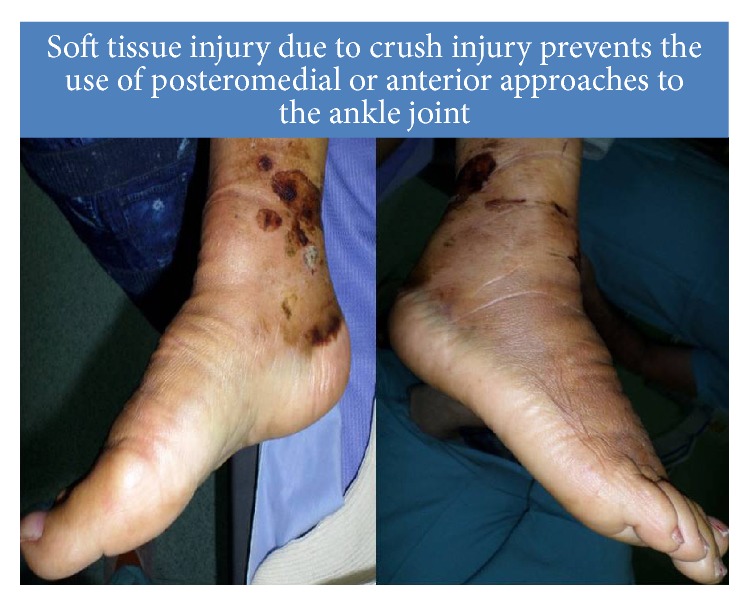
Following a crush of the leg, despite two weeks of elevation and compression therapy, the anterior and posteromedial approaches are fraught with risk due to extensive soft tissue injuries.

**Figure 2:**
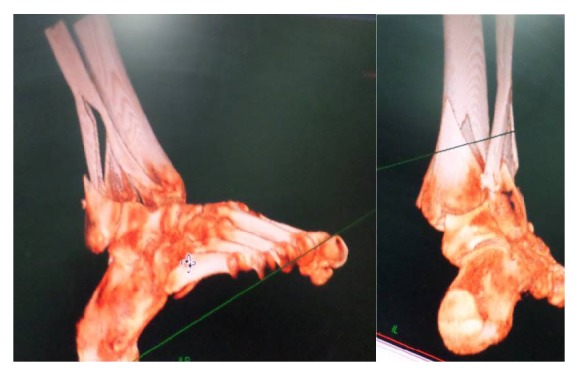
A pilon fracture with a major posterior malleolar fragment is seen. Note split of the fibula.

A 56-year-old patient was injured in a road accident with a major crush component. The leg was extremely swollen at admission and the skin was abraded over the anterior and medial aspects of the ankle (Figure 1). The patient was treated with limb elevation, an external compression pump, and compressive bandages for 14 days. A pilon fracture was diagnosed (Figure 2). A long delay due to extensive soft tissue injury precluded closed reduction and minimally invasive fixation of the large displaced posterior lip fragment.

**Figure 3:**
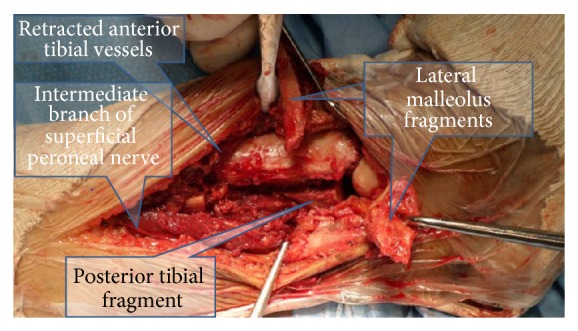
Transfibular approach to the lateral distal tibia. Note the intermediate branch of the superficial peroneal nerve as well as the anterior tibial vessels. The flipped-over lateral malleolus still retains the posterior (peroneal artery derived) blood supply.

The patient was operated on in a supine position, on a tilted table. A posterolateral approach was used, as this was the only area of intact skin. An osteotomy of the fibula was performed 7 cm above the distal fracture line (Figure 3). The syndesmosis was completely torn and the distal fragment was flipped over, thus allowing extensive approach to the distal tibia. The tibia was reduced under direct vision with internal fixation done by anteriorly placed screws via a minimally invasive technique. The fibula osteotomy and the fibular fracture were reduced and fixed using a plate. Bone grafting was not necessary due to excellent bone approximation. The medial malleolus was reduced and fixed using a plate inserted via a minimally invasive technique through a 3 cm oblique incision (due to the extensive skin damage). The postoperative course was uneventful. The patient was treated with a postoperative boot for one month and began partial weight bearing after 3 weeks. Bony union was obtained after 3 months.

## 3. Case Report 2

**Figure 4:**
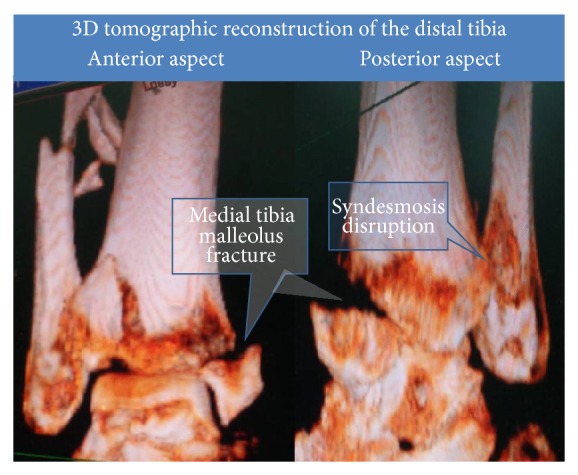
A pilon fracture with several major fragments is seen. The main fragment appears to be lateral accompanied by syndesmosis disruption.

A 54-year-old female fell down the stairs at her home. A pilon fracture was created with the distal tibial articular surface split into several fragments (Figure 4). The major articular fragment was displaced together with the lateral malleolus.

This type of fracture requires reduction of the lateral part of the distal tibia. However in this case it was not possible to perform closed reduction. Arthroscopic visualization confirmed complete disruption of the syndesmosis, with grade IV talar osteochondral fracture (20 mm^2^). The fragment was removed and microfractured. At this stage a lateral transfibular approach was utilized.

**Figure 5:**
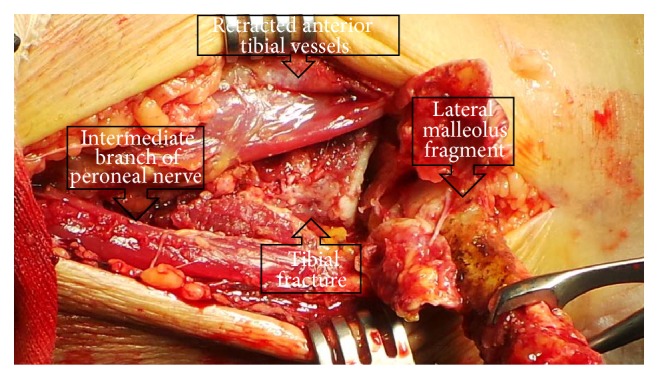
The lateral tibia is exposed via the transfibular approach. Note the proximity of the anterior tibial vessels and the deep peroneal nerve.

The fibular distal fragment was distally rotated (Figure 5), the tibial plafond fragment was fixed with two screws, and then the distal fibula was replaced in its notch. The fibular fracture was reduced with careful attention given to rotation and length restoration and was fixed using a locking plate. The medial malleolus and anterior plafond fragments were fixed by two percutaneously inserted screws.

## 4. Cadaver Study

The cadaver study was performed in the anatomical dissection lab at The Sackler School of Medicine (Tel Aviv University, Tel Aviv, Israel). Thirty-five specimens were dissected (14 females and 31 males; average age 78 years). The close proximity of the anterior tibial artery to the interosseous membrane overlies in the upper two-thirds of the leg; therefore, we attempted to define the relationship between the fibula and the anterior tibial artery and to define the retraction direction of the vessel.

The transfibular approach involves splitting or detaching the interosseous membrane from the bone. It is necessary to define the upper extent of the possible fibular osteotomy that does not jeopardize the anterior tibial artery. Notably, the deep peroneal nerve is located superficially to the anterior tibial artery. In the distal one-third of the leg, the nerve is located between the tibialis anterior and extensor hallucis longus muscles. Typically, the nerve passes deep into the extensor hallucis longus tendon and enters the interval between the extensor hallucis longus and extensor digitorum longus at an average distance of 12.5 mm proximal to the ankle. The deviation of the deep peroneal nerve from the interosseous membrane region to the space between the extensor digitorum longus and extensor hallucis occurs at an average distance of 5.8 ± 1.5 cm above the level of the tibial cartilage. The anterior lateral malleolar artery is a branch of the anterior tibial artery which arises at 17 ± 3 mm proximal to the distal tibial cartilage and should be sacrificed prior to rotation of the distal fibular. Nonetheless, blood supply to the distal aspect of the lateral malleolus is not compromised as there is also an arterial network originating from the posterior aspect. These branches stem from the peroneal artery. However in all cadavers examined an osteotomy performed less than 8 cm proximal to the tip of the lateral malleolus avoided damaging the superficial peroneal nerve.

## 5. Discussion

In tumor surgery, the advantage of the anterior approach is by preserving the integrity of the lateral ligaments of the ankle, whereas fibulectomy is superior in terms of access to posteriorly located distal tibial osteochondromata [2]. In trauma surgery the use of a fibulectomy is usually unnecessary unless an ankle fusion is performed [3, 4]. The two presented case reports are unusual due to the extensive soft tissue injury and the irregular type of tibial fracture; thus, a posteromedial or anterior approach to the tibia would be precarious. An anterior exposure of the ankle would prevent anatomic reduction of the posterior malleolus due to limited visibility as well as soft tissue contraction that would have precluded indirect reduction maneuvers.

It is possible to define a subgroup of ankle fracture cases with major distal tibia comminution that is mostly lateral, rather than the more common variant with medial comminution. This sort of fracture is preferably treated with a transfibular approach than with an anterior approach and it is possible to directly reduce the distal posterolateral fragment and reconstruct the fibular notch of the tibia.

Another indication for the transfibular approach is extensive lateral osteochondral lesion of the talus. In such cases, the commonly used approach via a transmedial malleolar osteotomy might not suffice and a transfibular approach could be utilized to gain extensive exposure to the talar dome or in rare cases the tibial plafond.

## 6. Recommended Surgical Technique

The patient is positioned on the unaffected side on a radiolucent table. An ipsilateral proximal leg or distal thigh tourniquet is advisable for hemostasis.

Incision placement is determined by the soft tissue conditions. Optimally, a direct lateral incision is made over the fibula, coursing approximately 10 cm from the distal tip to the fibular shaft. The incision might vary according to the soft tissue condition. After exposure of the distal fibula and the tibiofibular syndesmosis, the fibula is transected 5 cm proximal to the tip of the lateral malleolus, while preserving the soft tissue structures that attach to the posterior aspect of the fibula.

In cases of delayed reduction, it is likely that the syndesmosis is filled with scar tissue. In this case, the syndesmosis should be opened and the scar tissues removed. The fibula is flipped over distally with the rotation taking place over the intake distal soft tissue attachment; it is advisable to keep as much of the posterior soft tissue attachment as possible. This exposure allows excellent visualization of the lateral tibia and the fibular notch. Reduction of fracture fragments is performed under vision. The distal fibula is then flipped back into its anatomical position and the fracture is fixed with a lateral ankle locked plate. It is recommended that at least two syndesmotic screws are used to stabilize the syndesmosis. Syndesmosis reconstruction might be enhanced by placement of suture anchors into the distal tibia and fibula and knotting together the sutures.

During a transfibular approach with rotation of the distal lateral malleolar fragment, the syndesmosis is disrupted. Thus, such an approach requires syndesmotic restoration and fixation. In these 2 cases, two syndesmotic screws were used and the syndesmosis was reduced under direct visualization. Weight bearing can be commenced without removal of the syndesmotic screws which should be left indefinitely or at least for six months as suggested by Chu and Weiner [5].

There are two major risks in the transfibular approach. One is due to the close proximity of the anterior tibial vessels. Nonetheless, two vascular bundles enter the muscles at about 5 cm proximal to the distal tibia and at about 8 cm proximal to the distal tibia [6]; thus sacrificing one of these bundles is possible without significant clinical devascularization.

The other major risk is due to the proximity of the superficial peroneal nerve and, in particular, its intermediate branch, as well as the proximity of the deep peroneal nerve to the interosseous membrane. Utilizing the transfibular approach may damage the superficial peroneal nerve, due to its highly variable course [7].

Nerve injuries are likely to impair postoperative function and delay rehabilitation [6].

Nerve damage is unlikely if the fibular osteotomy/fracture is lower than 4.3 cm from the distal tibial cartilage. If the fracture is higher, as, for example, in the first patient case study described, then careful dissection of the nerve should be performed prior to rotation of the fibular fragment.

Sacrifice of the anterior lateral malleolar branch might facilitate mobilization of the anterior tibial vessels, subsequently protecting the deep peroneal nerve. The latter is usually located superficial to the vessels.

However, great care must be taken to avoid damage to the intermediate branch of the superficial peroneal nerve. If possible, the extensors should be mobilized together with the anterior tibial vessels in order to preserve blood supply to the muscles.

Nevertheless, a known variant of the intermediate branch of the superficial peroneal nerve may occur in which the branch passes over the fibula from the posterior to anterior aspect [8]. Such a variant was not encountered in the cadaver study performed.

In summary, although the transfibular approach is not a commonly used surgical approach to distal tibial fractures, knowledge of this approach adds an important option to the surgical arsenal.
